# Preparation of Activated Carbon From *Polygonum orientale* Linn. to Remove the Phenol in Aqueous Solutions

**DOI:** 10.1371/journal.pone.0164744

**Published:** 2016-10-14

**Authors:** Jia Feng, Shengli Shi, Liangyu Pei, Junping Lv, Qi Liu, Shulian Xie

**Affiliations:** School of Life Science, Shanxi University, Taiyuan, China; Northeast Forestry University, CHINA

## Abstract

Phenol components are major industry contaminants of aquatic environment. Among all practical methods for removing phenol substances from polluted water, activated carbon absorption is the most effective way. Here, we have produced low-cost activated carbon using *Polygonum orientale* Linn, a wide spreading species with large biomass. The phenol adsorption ability of this activated carbon was evaluated at different physico-chemical conditions. Average equilibrium time for adsorption was 120 min. The phenol adsorption ability of the *P*. *orientale* activated carbon was increased as the pH increases and reached to the max at pH 9.00. By contrast, the ionic strength had little effect on the phenol absorption. The optimum dose for phenol adsorption by the *P*. *orientale* activated carbon was 20.00 g/L. The dominant adsorption mechanism of the *P*. *orientale* activated carbon was chemisorption as its phenol adsorption kinetics matched with the pseudo-second-order kinetics. In addition, the equilibrium data were fit to the Langmuir model, with the negative standard free energy and the positive enthalpy, suggesting that adsorption was spontaneous and endothermic.

## Introduction

Phenol is one of human carcinogen with characteristic pungent smell and taste. Although with low solubility in water, low amount of phenol components could severely affect human and aquatic organisms’ gastrointestinal system and thus cause nausea, erythema, deep necrosis [1, 2]. Phenol substance is one of the major contaminants in the aquatic environment of from many modern industries, such as the coal conversion, dye manufacturing, papermaking, pesticide, petrochemical, pharmaceutical, and textile industries, etc [2]. It has been listed as one of the US Environmental Protection Agency priority pollutants [2, 3]. Attention has been increasingly concentrated on controlling phenol substances from industrial wastewater for decades, and limits have been placed on the amounts of phenol discharged, to attempt to ameliorate the direct and potentially negative impacts of phenol on the aquatic environment [4, 5]. There are various chemical, physical, physicochemical, and biological methods used to remove phenol from wastewater [6, 7], such as distillation [8], extraction with membrane adsorption [9], microbial degradation [10], and oxidation using ozone [11], hydrogen peroxide, and chlorine oxides [7]. Among all these methods, adsorption has been proven to be the most effective and economic way for removing phenol from wastewater [2]. Activated carbon is the best and most common choice, which possesses excellent phenol adsorption ability [12–14]. Comparing to traditional physical activation for generating activated carbon, chemical activation presents several advantages, including single-step activation, low activation temperatures, low activation time, and better porous structure [15]. Thus, numerous attempts have been done to produce low-cost activated carbon for use in adsorptive dephenolation processes using a wide range of agricultural waste products[2, 16], such as nutshells, wood, fruit stones and so on. The agricultural wastes, including corn cobs, banana piths, oil palm, have been utilized as a precursor for the preparation of activated carbon [2, 3, 17].

*Polygonum orientale* Linn. is an annual herbage commonly occurred in freshwater wetlands [18, 19]. It grows rapidly and produces large quantities of biomass, which is usually used to construct wetlands for wastewater treatment [17]. This plant has porous caudex system and the texture is between medium hard to soft. Its mass of carbon is about 40–45%, volatiles mass is 58–60%, and the density is 0.50–0.60 cm^3^/g. Thus, it could be considered to produce an effective activated carbon [20]. The potential capabilities of *P*. *orientale* in phytoremediation, especially of phenol, have been little reported [16].

In this study, we aimed to evaluate the potential for using activated carbon produced from *P*. *orientale* to remove phenol in aqueous solutions. The influences of several operating parameters, such as the initial phenol concentration, adsorbent dose, initial solution pH, and temperature, on the adsorption of phenol were examined in batch tests. Pseudo-first-order, pseudo-second-order, and diffusion kinetics models were used to identify the possible mechanisms involved in the adsorption process, and the Langmuir and Freundlich equations were used to analyze the adsorption equilibrium.

## Materials and Methods

### Preparation of *Polygonum orientale*

The *Polygonum orientale* was collected from Fenhe, Shanxi Province, China. The sampling site is a public area and no specific permission was required. And there is no endangered or protected species in our sampling area. The material was cut into small pieces of 2–3 cm and washed with distilled water to remove the water soluble impurities. Then they were dried in an air drying oven (101A-3, Suoyu, Shanghai, China) at 80°C for 6 h to get rid of the moisture and other volatile. The dried material was grounded into powder using an HR-1727 grinder (Philips, Zhuhai, China) and sieved through standard sieves to give 70–100 μm mesh particles.

The Brunauer Emmett Teller (BET) surface area and pore characteristics of *P*. *orientale* were measured by the surface area and pore analyzer (ASAP2020, Micromeritics, USA), under nitrogen adsorption at 77K.

The infrared spectroscopy was determined wave number from 400–4,000 cm^-1^ by Fourier transforms infrared (FTIR) spectrometer (AVATAR 370, Thermo Nicolet, USA). Consequently, the numbers of surface groups on *P*. *orientale* were determined by titration method [21].

### Adsorption experiments

Stock solution containing phenol at a concentration of 1000 mg/L was prepared. Experimental solutions with gradient concentrations of phenol were prepared by diluting the stock solution. The adsorption effects at different conditions (contact times, shaking rates, temperatures, solution pH values, and ionic strengths) were examined. The experiments were performed using a temperature-controlled water bath shaker (HH-2, Guohua, Beijing, China). The initial pH of each experimental solution was adjusted to the desired value by adding HCl_(aq)_ or NaOH_(aq)_. The ionic strength of each solution was adjusted by adding NaCl and MgCl_2_.

In brief, the adsorption experiments were performed by shaking 2.00 g of *P*. *orientale* powder with 100 mL of the experimental solution containing a known concentration of phenol in the temperature-controlled water bath shaker. The mixture was continuously mixed, with a constant agitation speed of 120 rpm (using a 78–1 mixing instrument, Jintanhuanyu, Jiangsu, China). After the absorpting, the solution was filtered and the remaining phenol concentration was determined by OD_270_ using UV/visible spectrophotometer (SP-752PC, Guangpu, Shanghai, China). The phenol uptake at equilibrium *q*_*e*_ (mg/g) and the percentage of phenol removed from the solution (Adsorption%) were calculated using Eqs (1) and (2), respectively,

$$q_{e}=\frac{(C_{0}-C_{e})V}{W},$$ (1)

$$Adsorption(\%)=\frac{C_{0}-C_{e}}{C_{0}}\times 100,$$ (2)

where *C*_*0*_ and *C*_*e*_ (mg/L) are the initial and equilibrium phenol concentrations, respectively, *V* (L) is the volume of the solution, and *W* (g) is the mass of adsorbent used.

### Adsorption kinetics model

Two different kinetics models were applied to the experimental data to allow the kinetics of the adsorption of phenol onto the *P*. *orientale* powder to be evaluated.

#### Pseudo-first-order model

The pseudo-first-order equation Eq (3) has been widely used to describe the adsorption of an adsorbate in an aqueous solution onto an adsorbent. This equation is based on the assumption that the rate of change in the uptake of a solute by an adsorbent over time is directly proportional to the change in the difference between the saturation concentration and the amount of solute that is adsorbed over time [22].

$$\frac{{dq}_{t}}{dt}=k_{1}(q_{e}-q_{t})$$ (3)

When *q*_*t*_ *=* 0 at *t* = 0, Eq (3) can be integrated to give Eq (4),

$$\log\;\left(q_{e}-q_{t}\right)=\log\;q_{e}-\frac{k_{1}}{2.303}t,$$ (4)

in which *q*_*e*_ and *q*_*t*_ (mg/g) are the amounts of phenol adsorbed at equilibrium and at time *t*, respectively, *t* (min) is the contact time, and *k*_*1*_ (1/min) is the rate constant for this equation. The values of *k*_*1*_ and *q*_*e*_ can be calculated from a plot of log (*q*_*e*_−*q*_*t*_) against *t*.

#### Pseudo-second-order model

The pseudo-second-order kinetics equation [23] can be presented as shown in Eq (5).

$$\frac{{dq}_{t}}{dt}=k_{2}{\left(q_{e}-q_{t}\right)}^{2}$$ (5)

On integrating Eq (5) and noting that *q*_*t*_ = 0 at *t* = 0, the equation can be rearranged into the linear form shown in Eq (6),

$$\frac{t}{q_{t}}=\frac{1}{k_{2}{q_{e}}^{2}}+\frac{1}{q_{e}}t,\;where\;h=k_{2}{q_{e}}^{2},$$ (6)

where h (mg/(g min)) is the initial adsorption rate and *k*_*2*_ (g/(mg min)) is the pseudo-second-order rate constant. The values of *q*_*e*_, *k*_*2*_, and *h* can be obtained from a linear plot of *t*/*q*_*t*_ against *t*.

### Adsorption mechanism

The diffusion mechanism could not be identified using the pseudo-first-order and pseudo-second-order kinetics models. The intraparticle diffusion model was therefore used to gain insight into the mechanisms involved and to identify the rate-controlling step. This model described by Weber and Morriss [24], shown in Eq (7), can be used to gain insight into the mechanisms and rate-controlling steps that affect adsorption kinetics.

$$q_{t}=k_{pi}t^{1/2}+C_{i}$$ (7)

In Eq (7), *k*_*pi*_ (mg/ (g min^1/2^)) is the intraparticle diffusion rate constant for stage *i* and *C*_*i*_ is the intercept for stage *i*. The *C* value gives information on the thickness of the boundary layer, with a larger intercept meaning that the boundary-layer effect is stronger. To use the intraparticle diffusion model, a plot of *q*_*t*_ against *t*^1/2^ should give a straight line, with a slope of *k*_*p*_ and an intercept of *C* [14, 25].

### Adsorption isotherms

The adsorption isotherm model is of fundamental importance to the description of the interactive behavior between an adsorbate and an adsorbent. The analysis of isotherm data is an important way for predicting the adsorption capacity and describing the surface properties of an adsorbent and the affinity of the adsorbent for the adsorbate of interest. The Langmuir and Freundlich isotherms were used to analyze the experimental equilibrium data for the sorption of phenol by the *P*. *orientale* powder in our study.

#### Langmuir isotherm

The Langmuir isotherm is based on the assumption that the adsorbent has a homogeneous structure and that all sorption sites are identical and energetically equivalent [26]. The Langmuir isotherm equation can be written as

$$\frac{C_{e}}{q_{e}}=\frac{1}{Q_{m}b}+\frac{1}{Q_{m}}C_{e},$$ (8)

where *C*_*e*_ (mg/L) is the equilibrium concentration of the adsorbate, *q*_*e*_ (mg/g) is the amount of adsorbate adsorbed per unit mass of adsorbent, *b* (L/mg) is the Langmuir adsorption constant, and *Q*_*m*_ (mg/g) is the maximum amount adsorbed. A dimensionless constant separation factor or equilibrium parameter *R*_*L*_ can be defined using Eq (9) [27] to determine if adsorption is favorable.

$$R_{L}=\frac{1}{1+bC_{0}}$$ (9)

In Eq (9), *b* (L/mg) is the Langmuir isotherm constant and *C*_*0*_ (mg/L) is the initial phenol concentration. The *R*_*L*_ value indicates whether the isotherm is favorable (0<*R*_*L*_<1), unfavorable (*R*_*L*_>1), linear (*R*_*L*_ = 1), or irreversible (*R*_*L*_ = 0). The Langmuir constants can be obtained from a plot of *C*_*e*_/*q*_*e*_ against *C*_*e*_.

#### Freundlich isotherm

The Freundlich isotherm can be used to describe a heterogeneous system and reversible adsorption that is not restricted to the formation of a monolayer [28]. The Freundlich isotherm can be expressed in a linear form as

$$\log\;q_{e}=\log\;K_{F}+\left(\frac{1}{n}\right)\;\log\;C_{e},$$ (10)

where *K*_*F*_ and *n* are Freundlich constants. *K*_*F*_ ((mg/g) (L/mg)^1/*n*^) indicates the adsorption capacity of the adsorbent. A larger *K*_*F*_ value indicates that the adsorbent has a larger adsorption capacity. The value of *n* indicates how favorable the adsorption process is. A value of 1/*n<*1 indicates a normal Friedrich isotherm and cooperative adsorption. The Freundlich constants can be obtained from a plot of log *q*_*e*_ against log *C*_*e*_.

### Adsorption thermodynamics

The change in standard free energy (Δ*G*; kJ/mol), enthalpy (Δ*H*; kJ/mol), and entropy (Δ*S*; kJ/(mol K)) can be determined using Eqs (11) and (12) [29].

ΔG=−RTlnK (11)

ΔG=ΔH−TΔS (12)

In these equations, *R* is the universal gas constant (8.314 kJ/(mol K)), *T* is the temperature (K), and *K* is the Langmuir constant (L/mol) obtained from a plot of *C*_*e*_/*q*_*e*_ against *C*_*e*_. The Δ*H* and Δ*S* values can be calculated from the slope and intercept of the line found by plotting Δ*G* against *T*.

## Results and Discussion

### Characterization of *Polygonum orientale*

The characteristics of the *Polygonum orientale* particle were shown in Table 1. The BET surface area was 1255.10 m^2^/g, which was much higher than other various low cost activated carbons reported in literature [3, 30–32]. The BJH adsorption average pore diameter was 7.433 nm and the BJH adsorption average pore volume was 0.337 cm^3^/g. For the pore analysis, the good surface area and the mesopore volume are an indication of effective adsorption of phenol on the adsorption.

**Table 1:** Chemical characteristics of *Polygonum orientale*.

Sample	pH_pzc_	Total basicity (mmol/g)	Total acidity (mmol/g)	Carboxyls (mmol/g)	Phenols (mmol/g)	Lactones (mmol/g)	Carbonyl (mmol/g)
***P*. *orientalis***	7.10	1.5300	1.5273	0.3167	0.7086	0.2000	0.3020

**Fig 1:**
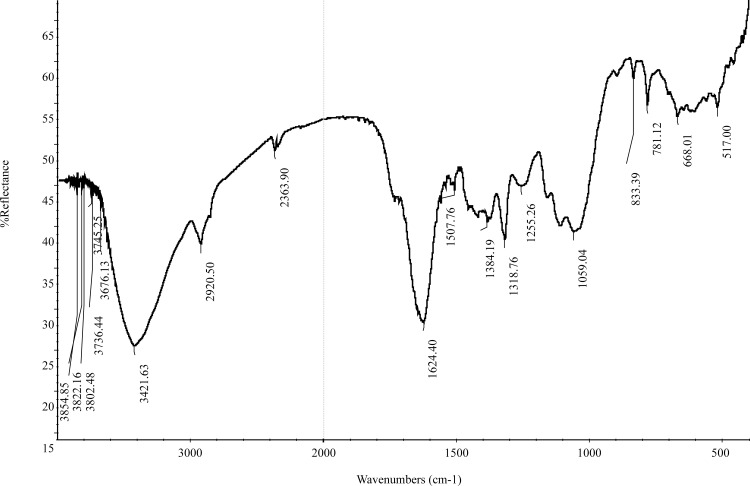
Fourier transform infrared spectra on *Polygonum orientale* surface.

According to the FTIR spectroscopy data, many surface organic functional groups of *P*. *orientale* have been identified (Fig 1), which was supposed to be responsible for phenol uptake. The characteristic band at 3421.63 cm^-1^ could be assigned to the–OH stretching vibration of hydroxyl functional groups including hydrogen bonding [33, 34]. The band at around 2920.50 cm^-1^ was ascribed to the asymmetric C–H stretching vibrations in aliphatic structure. The peaks occurring at 2363.90 cm^-1^ was due to C–O vibrations [2]. The band located at 1624.40 cm^-1^ was suggested to be C = C stretching in ethylene groups. The band at 1507.760 cm^-1^ was due to C = C stretching in aromatic group [2]. The peak occurring at 1384.19 cm^-1^ was ascribed to the C–H stretching. The band located at 1318.76 cm^-1^ represented C–O vibration in carboxylate group. The band at around 1255.26 cm^-1^ was due to the C–OH stretching vibrations in alcoholic groups and carboxylate group. The peak at 1059.04 cm^-1^ was ascribed C–H of the alcoholic groups and the carboxylic acids [24]. The band at around 833.39 (also 781.12) cm^-1^ was assigned to C–H twist vibrations in aromatic groups. The band located at 668.01 cm^-1^ attributed to O–H bending vibrations in alcohol and phenol groups. As stated by the possible functional groups assigned to the peak in FTIR spectra, the oxygen groups were the important functional groups of *P*. *orientale*, which included ethers, esters, alcohols and phenol groups. In order to further clarify the chemistry portraits of the *P*. *orientale*’s surface, Boehm titration has been done (Table 1). *P*. *orientalis* has general equal acidic groups and basic ones on the surface. Thus, it could be considered that the *P*. *orientalis* activated carbon is neutral.

### Effect of the initial phenol concentration, time, and temperature

**Fig 2:**
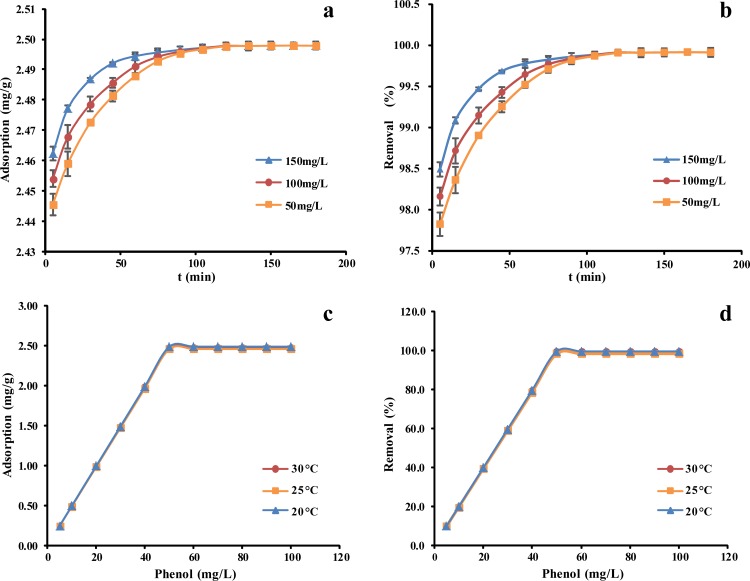
(a, b) Effect of adsorption equilibrium time and comparison (temperature = 25±1°C), (c, d) equilibrium temperature and comparison of different phenol concentrations on *P*. *orientale* (*P*. *orientale* dosage = 20.00 g/L).

The change in the amount of phenol adsorbed by the *P*. *orientale* powder over time (up to 200 min) was investigated. Phenol adsorption is significantly influenced by the initial concentration of phenol in aqueous feed solution. The higher initial concentration was used, the shorter time was taken to reach the equilibrium. Nevertheless, the equilibrium time for the lowest concentration, 50 mg/L, being tested in this study was 120 min. Therefore, the equilibrium time in our study was considered to be 120 min, as this time duration was sufficient for equilibrium to be reached at all three concentrations, 50 mg/L, 100 mg/L, and 150 mg/L (Fig 2A and 2B). As shown in the figure, the amount of phenol adsorbed increased as the initial phenol concentration increased in the first 100 min at room temperature. The rapid uptake at initial phase is due to the vacant adsorption sites making it easier for phenol interaction with these sites [35]. And in the initial phase, transport to the sites is also important for a reaction to be fast. The amount of phenol uptake remained constant after 120 min. The adsorption amount increased with an increase in initial phenol concentration. It was due to that higher initial phenol concentration would have stronger driving force for the transferring the phenol from the aqueous phase to the solid phase and more collisions has occurred between phenol ions and the adsorbent surfaces [36].

The temperature variation is another important factor affecting the activated carbon adsorption. Since adsorption is an exothermic process, it is suggested that adsorption capacity normally decreases when temperature goes up [12]. However, the adsorption capacity of *P*. *orientale* activated carbon showed no changes at all three temperature conditions tested in this study (Fig 2C and 2D). As all three conditions were considered as room temperature, it could be concluded that the room temperature was the best option for adsorption by *P*. *orientale* activated carbon and little effects on the adsorption ability by temperature changing at room temperature range.

### Effect of adsorbent dose

**Fig 3:**
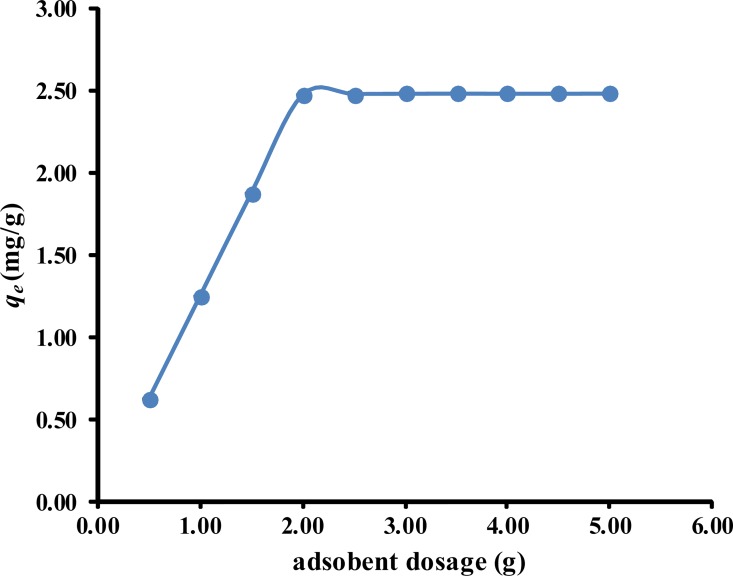
Effect of adsorbent dosage on adsorption of phenol by *P*. *orientale* (C_0_ = 50 mg/L; *P*. *orientale* dosage = 20.00 g/L; temperature = 25±1°C).

The influence of the adsorbent dose on the amount of phenol removed by the *P*. *orientale* powder is shown in Fig 3. The percentage of phenol removed from the solution increased from 25% to 98% as the *P*. *orientale* dose was increased from 5.00 g/L to 20.00 g/L, and then remained almost constant at higher doses of *P*. *orientale*. This was expected because the adsorbed phenol either blocked access to the initial pores or caused particle aggregation, thereby reducing the available active sites. Increasing the adsorbent dose has increased the surface area of the adsorbent in the test solution and thus the availability of adsorption sites [37]. The optimum *P*. *orientale* dose was found to be 20.00 g/L, as no obvious increase adsorption was seen with absorbent dose higher than 20.00 g/L.

### Effect of pH

**Fig 4:**
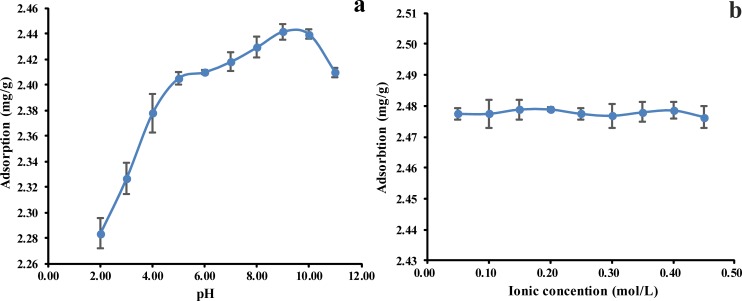
(a) Effect of pH value on adsorption of phenol by *P*. *orientale*, (b) ionic concentration on adsorption of phenol by *P*. *orientale* (C_0_ = 50 mg/L; *P*. *orientale* dosage = 20.00 g/L; temperature = 25±1°C).

The effect of the pH on phenol clearance efficiency was assessed by absorption experiments at gradient pH conditions, from pH 2.00 and pH 12.00 (Fig 4A). The adsorption capacity for phenol increased as the initial pH was increased from a value of 2.00 to a value of 9.00, and the percentage of the phenol that was removed from the solution increased dramatically, from 91.30% to 96.30%. However, the percentage of the phenol that was removed decreased as the pH was increased from a value of 9.00 to a value of 11.00.

There were a great number of critical functional groups on the *P*. *orientale* surface according to Fig 1. The pH of phenol sorption from the aqueous depended on the type and ionic state of the functional groups and the phenol chemistry [2]. Both the phenol and the surface groups coexist in their protonated and deprotonated forms without buffered solutions, depending on the p*K*_a_ (= 9.89) values of phenol [12]. The phenol adsorption was decreased from pH 9.00 to pH 2.00, because of the decreased H^+^ adsorption on the carbonyl sites and suppressed phenol adsorption on these sites. At the acidic pH, both the functional groups on the carbon surface and the phenolic compounds were in the non-ionized forms, pH<p*K*_a_. The surface groups are either neutral or positively charged. As the initial solution pH increased, the number of negatively charged active sites increased while the number of positively charged sites decreased. The strength of the electrostatic repulsion between an adsorbent site and a positively charged phenol ion therefore decreased, which could result in more adsorption occurring. Less phenol has been adsorbed by the *P*. *orientale* powder at acidic pH values because of competition between the excess hydrogen ions in the solution and the *P*. *orientale* ions available as adsorption sites [32, 38, 39]. At weak alkaline pH values, the presence of OH^−^ ions promotes the transformation of the phenol molecules into the ionic state. This could explain the highest adsorption capacity and highest adsorption rate found at weakly alkaline pH values (i.e., at relatively low OH^−^ concentrations). At a value pH = 11.00 (for pH>pKa), the phenol dissociates, and forms phenolate anions, while the surface functional groups are either neutral or negatively charged. The electrostatic repulsion between the *P*. *orientale* material and the phenol ions would lead to the decrease of the amount of phenol adsorbed [32, 40].

### Effect of ionic strength

The effect of changing the ionic strength of the solution on the amount of phenol adsorbed was measured, and plots of the amounts of phenol adsorbed against the NaCl concentrations used are shown in Fig 4B. The amount of phenol adsorbed by *P*. *orientale* and the adsorption rate did not vary in any clear way as the ionic strength was changed. As described by Lützenkirchen [41], two different surface complexes, inner-sphere and outer-sphere complexes, can form during the sorption process. In inner-sphere surface complexes covalent bonds form between the adsorbed molecules or ions and the surface functional groups, whereas no covalent bonds form in outer-sphere surface complexes. Non-covalent interactions, such as electrostatic attraction, hydrogen bonding, or hydrophobic attraction, are therefore responsible for the adsorption of adsorbate species in outer-sphere complexes. It has been assumed that insensitivity to the ionic strength indicates that inner-sphere surface complexes are formed, and that a decrease in the amount of adsorbate adsorbed as the ionic strength increases indicates that outer-sphere surface complexes are formed. The independence of the adsorption of phenol by the *P*. *orientale* material in our study could therefore be explained by inner-sphere complexes forming between the phenol and the *P*. *orientale* material [42, 43].

### Adsorption kinetics

**Fig 5:**
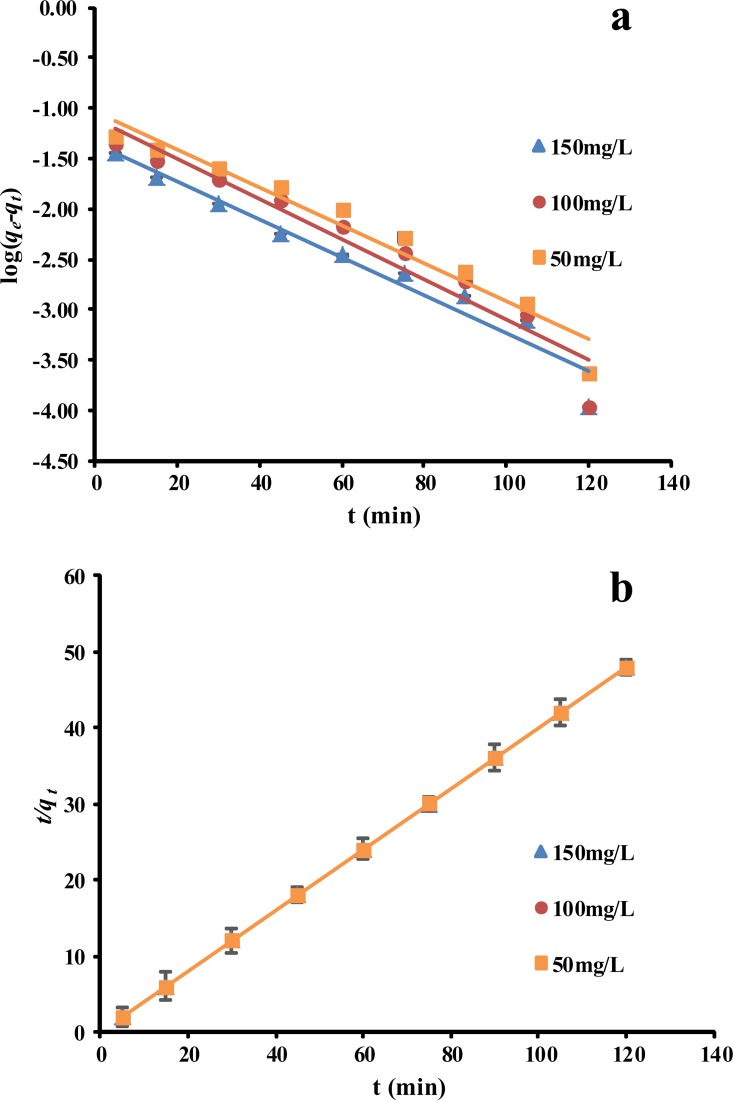
(a) Pseudo-first-order kinetics plots on adsorption of phenol by *P*. *orientale*, (b) Pseudo-second-order kinetics plots on adsorption of phenol by *P*. *orientale* (C_0_ = 50 mg/L; *P*. *orientale* dosage = 20.00 g/L; temperature = 25±1°C).

The kinetics parameters found for the adsorption of phenol by the *P*. *orientale* material are shown in Tables 2 and 3. The *R*^*2*^ value obtained for the pseudo-first-order kinetics model was relatively low, and the calculated *q*_*e*_ (cal) values were much lower than the experimental *q*_*e*_ (exp) values (Fig 5A). However, the experimental results fitted the pseudo-second-order model well, with an extremely high *R*^*2*^ value of 0.9999 (i.e., close to unity). Moreover, the experimental *q*_*e*_ (exp) values agreed well with the calculated values (Fig 5B). These results indicate that the adsorption of phenol by the *P*. *orientale* material followed the pseudo-second-order kinetics model. This means that the adsorption mechanism could depend on the concentrations and characteristics of both the adsorbate and the adsorbent [44], and that the rate limiting step might be chemisorption involving valence forces caused by the sharing or exchange of electrons [43, 45].

**Table 2:** Pseudo-first-order and pseudo-second-order kinetic equation.

*C*_*e*_ (mg/L)	Pseudo-first-order		Pseudo-second-order	
**50**	y = -0.0188 x—1.0335	R^2^ = 0.9540	y = 0.3996x + 0.1101	R^2^ = 0.9999
**100**	y = -0.0199 x—1.1062	R^2^ = 0.9347	y = 0.3998 x + 0.0839	R^2^ = 0.9999
**150**	y = -0.0188 x—1.3461	R^2^ = 0.9537	y = 0.3999 x + 0.0512	R^2^ = 0.9999

**Table 3:** Pseudo-first-order and pseudo-second-order kinetic parameters.

*C*_*e*_ (mg/L)	Pseudo-first-order model				Pseudo-second-order model	
	*q*_*e*_(exp) (mg/g)	*k*_1_×10^−2^ (min^-1^)	*q*_*e*_(cal) (mg/g)	*k*_2_ (g/mg^.^min)	*q*_*e*_(cal) (mg/g)	*h* (mg/g^.^min)
**50**	2.4979	4.3189	0.0926	1.4499	2.5024	9.0793
**100**	2.4979	4.5967	0.0783	1.9054	2.5015	11.9223
**150**	2.4979	4.3298	0.0451	3.1236	2.5001	19.5239

### Adsorption mechanism

The diffusion mechanism could not be identified using the pseudo-first-order and pseudo-second-order kinetics models. The intraparticle diffusion model was therefore used to gain insight into the mechanisms involved and to attempt to identify the rate-controlling step.

**Fig 6:**
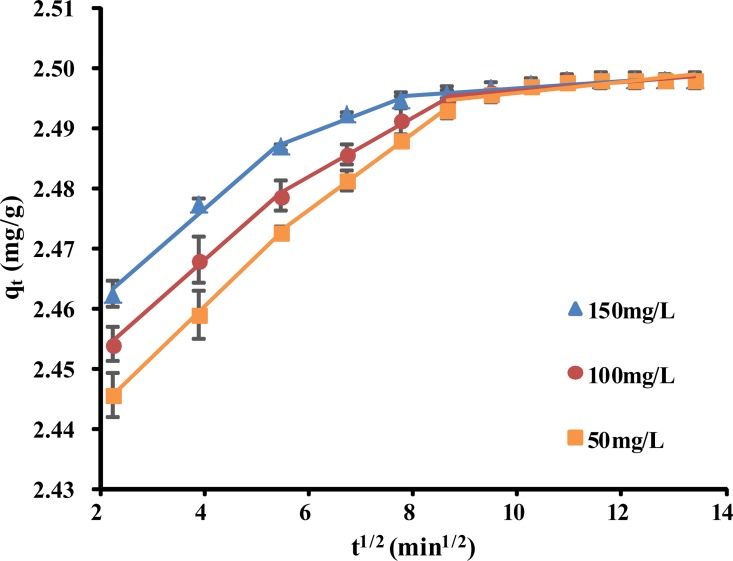
Intraparticle diffusion plots for adsorption of phenol on by *P*. *orientale* (C_0_ = 50, 100, 150 mg/L; *P*. *orientale* dosage = 20.00 g/L; temperature = 25±1°C).

The amounts of phenol adsorbed compared with the *t*^*1/2*^ values for the intraparticle transport of phenol by *P*. *orientale* for different initial phenol concentrations are shown in Fig 6. The plots are multilinear with three portions, each of which is for a different stage in the adsorption process. The first portion, with the steepest slope, is the external mass transfer stage. The second portion is the gradual adsorption stage, in which intraparticle diffusion is the rate-limiting step. The third portion is the final equilibrium stage, in which intraparticle diffusion starts to slow down because of the extremely low adsorbate concentration remaining in the solution [32, 46]. The lines do not pass through the origin, so we can conclude that intraparticle diffusion is not the only rate-limiting step and that boundary layer control may be involved in the process [47]. Some other mechanisms, such as the formation of complexes or ion exchange, may also exert some control over the adsorption rate [48]. The model parameters defined in the equation described are given in Table 4. The *C* values increased as the initial phenol concentration increased and *K*_*pi*_ decreased. This indicates that the rate at which phenol was removed was higher at the beginning of the adsorption tests because of the large surface area of the adsorbent that was available to adsorb phenol.

**Table 4:** Intra-particle diffusion parameters.

*C*_*0*_(mg/L)	Intra-particle diffusion model								
	*k*_*p1*_ (mg/gmin^1/2^)	*k*_*p2*_ (mg/gmin^1/2^)	*k*_*p3*_ (mg/gmin^1/2^)	*C*_*1*_	*C*_*2*_	*C*_*3*_	(*R*_*1*_)^2^	(*R*_*2*_)^2^	(*R*_*3*_)^2^
**50**	0.0083	0.0064	0.0009	2.4269	2.438	2.4863	0.9999	0.9957	0.7301
**100**	0.0086	0.005	0.0007	2.4375	2.452	2.4894	0.9954	0.9901	0.7394
**150**	0.0076	0.0034	0.0006	2.4462	2.4684	2.4907	0.9859	0.9695	0.833

### Adsorption isotherms

**Fig 7:**
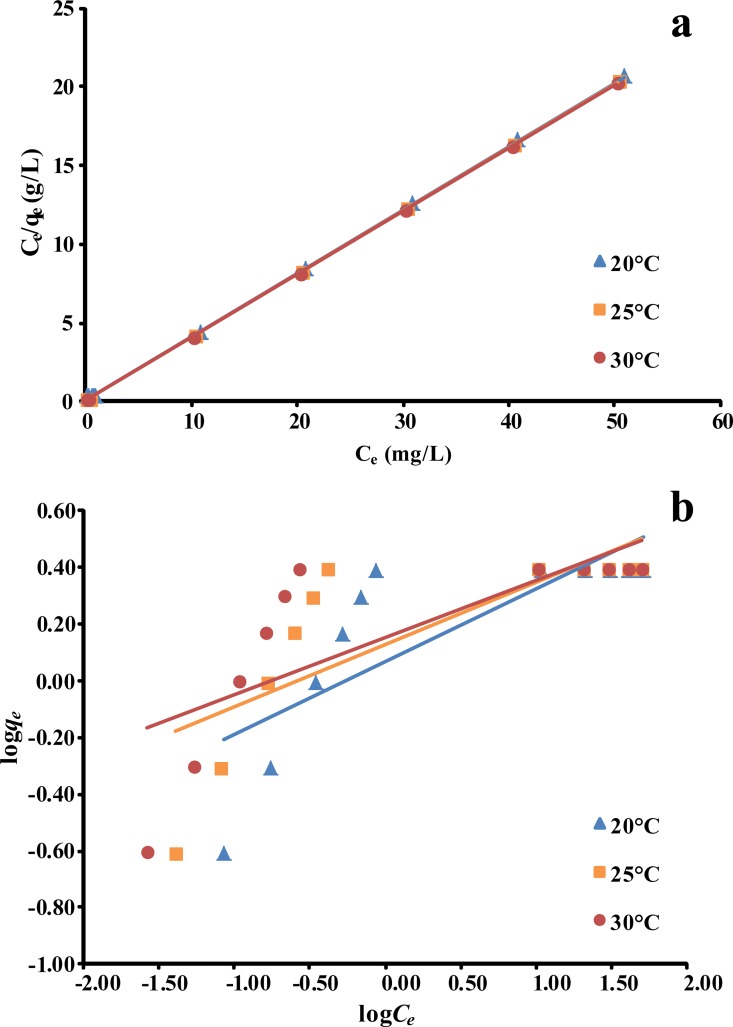
(a) Langmuir isotherm for adsorption of phenol onto *P*. *orientale* at different temperatures, (b) Freundlich isotherm for adsorption of phenol onto *P*. *orientale* at different temperatures (temperature = 20, 25, 30°C; C_0_ = 5–100 mg/L; *P*. *orientale* dosage = 20.00 g/L)

Plots of *q*_*e*_ against *C*_*0*_ for the adsorption of phenol onto *P*. *orientale* at 20°C, 25°C, and 30°C are shown in Fig 7A and 7B, according to the Langmuir and Freundlich isotherms, respectively. The values for the constants calculated using the two isotherms are given in Tables 5 and 6. The adsorption of phenol onto *P*. *orientale* fitted the Langmuir isotherm model well, giving high *R*^*2*^ values (0.9998–0.9999). This may have been caused by the homogeneous distribution of active sites on the surfaces of the *P*. *orientale* material. Furthermore, the *R*_*L*_ values for the Langmuir isotherm were between zero and one, but the Freundlich constant 1/*n* was lower than one, indicating that adsorption was favorable. The maximum adsorption capacity *Q*_*m*_ increased as the temperature increased, showing that the process was endothermic. The high adsorption capacity found in this study revealed that *P*. *orientale* is a promising adsorbent for removing phenol from aqueous solutions.

**Table 5:** Langmuir and Freundlich isotherm equation.

T (K)	Langmuir equation		Freundlich equation	
**293**	y = 0.4030 x + 0.1501	R^2^ = 0.9999	y = 0.2597 x + 0.0679	R^2^ = 0.6229
**298**	y = 0.4014 x + 0.0702	R^2^ = 0.9999	y = 0.2199 x + 0.1271	R^2^ = 0.5920
**313**	y = 0.4009 x + 0.0454	R^2^ = 0.9999	y = 0.2015 x + 0.1531	R^2^ = 0.5770

**Table 6:** Isotherm model constants of four isotherm models for phenol adsorption onto *P*. *orientale*.

T (K)	Langmuir model parameters			Freundlich model parameters	
	*Q*_*m*_ (mg/g)	*b* (L/mg)	*R*_*L*_	*K*_*F*_ (mg/g(L/mg)^1/n^)	*1/n*
**293**	2.4816	16.5277	0<*R*_*L*_<1	1.1692	0.2597
**298**	2.4912	35.4996	0<*R*_*L*_<1	1.3399	0.2199
**313**	2.4943	54.9778	0<*R*_*L*_<1	1.4227	0.2015

### Adsorption thermodynamics

The adsorption thermodynamics results are given in Table 7. A positive Δ*H* value was found, further confirming that the adsorption process was endothermic. Δ*G* was negative, reflecting the spontaneity and feasibility of the adsorption process.

**Table 7:** Thermodynamic parameters for the adsorption of phenol onto *P*. *orientale* at different temperatures.

T (K)	Thermodynamic equation: y = -162.59x + 40294, R^2^ = 0.8827		
	ΔG (kJ/mol)	ΔH (kJ/mol)	ΔS (kJ/mol K)
**293**	-6.833	40.294	-0.162
**298**	-8.843		
**313**	-10.427		

## Conclusions

The present work showed that *P*. *orientale* could be used as an good adsorbent for the removal of phenol in aqueous solutions. The amount of phenol adsorbed depended strongly on the contact time, pH, adsorbent dose, and temperature, but not ionic strength. More phenol was adsorbed as the pH increased, the optimum being pH 9.0. The optimum adsorbent dose was 20.0 g/L. Equilibrium was reached in 120 min. Adsorption followed pseudo-second-order kinetics, so the dominant process was chemisorption. The equilibrium data were well described by the Langmuir model. Δ*G* was negative and Δ*H* was positive, meaning adsorption was spontaneous and endothermic. There is great potential for using *P*. *orientale* as an economical and efficient adsorbent for removing phenol from aqueous solutions.
